# Successful thoracic duct identification with patent blue V during thoracic duct ligation for chylothorax: a case report

**DOI:** 10.1186/s44215-022-00010-5

**Published:** 2022-11-01

**Authors:** Yamato Suzuki, Koji Yamana, Hisato Ishizawa, Hiroshi Kawai, Yasushi Matsuda, Ryoichi Kato, Yasushi Takagi, Yasushi Hoshikawa

**Affiliations:** 1 Department of Thoracic Surgery, Fujita Health University School of Medicine, 1-98 Dengakugakubo, Kutsukake-cho, Toyoake, Aichi 470-1192 Japan; 2 Department of Cardiovascular Surgery, Fujita Health University School of Medicine, 1-98 Dengakugakubo, Kutsukake-cho, Toyoake, Aichi 470-1192 Japan; 3 Department of Radiology, Fujita Health University School of Medicine, 1-98 Dengakugakubo, Kutsukake-cho, Toyoake, Aichi 470-1192 Japan

**Keywords:** Chylothorax, Thoracic duct ligation, Patent blue, Video-assisted thoracic surgery

## Abstract

**Background:**

Chylothorax after thoracic surgery is a rare but severe complication. When thoracic duct ligation is performed for chylothorax, identification of the leakage site and the thoracic duct course is necessary. Administering milk orally or through a nasogastric tube and injecting indocyanine green into lymph nodes and lymphatic vessels can be performed to identify the leakage site and the thoracic duct course. However, the injection of patent blue V into the inguinal lymph nodes has not been reported.

**Case presentation:**

A 69-year-old man underwent aortic replacement surgery for an aortic aneurysm of the distal arch. On postoperative day 3, after resuming oral intake, the patient was diagnosed with chylothorax. The patient was treated with fasting and total parenteral nutrition, but the chylous pleural effusion continued at 500–1000 ml daily. A plan for thoracic duct ligation was made. We injected patent blue V into the inguinal lymph node to identify the leakage site and the thoracic duct course. The blue-stained thoracic duct was identified and ligated, but the leakage site could not be identified because of the surrounding lung adhesions. The thoracic drain was removed on day 6 post-second operation, and the chylothorax did not recur.

**Conclusion:**

Identifying the thoracic duct course using patent blue V is useful during thoracic duct ligation for chylothorax.

## Background

Chylothorax is a rare but severe complication of thoracic surgery. Treatments for chylothorax includes a low-fat medium-chain triglyceride diet, fasting, total parenteral nutrition (TPN), octreotide acetate, pleurodesis, thoracic duct embolization, and thoracic duct ligation (TDL) [1–5]. The treatment method is selected according to the drainage volume of the chylous pleural effusion and the patient's condition. When performing TDL, it is necessary to identify the leakage site of the chylous pleural effusion and thoracic duct (TD) course intraoperatively. Administering a high-fat diet, such as milk or olives orally or through a nasogastric tube during TDL, has been reported [3, 4]. Indocyanine green (ICG) injection into lymph nodes and vessels has also been performed [5, 6].

Patent blue V is used during lymphangiography to stain lymph nodes and vessels blue. It can also identify sentinel lymph nodes in breast cancer [7]. However, its use for identifying the leakage site and the TD course during TDL has not been reported. We report a case in which the TD course was identified using patent blue V during TDL for chylothorax after surgery for a thoracic aortic aneurysm.

## Case presentation

**Fig. 1:**
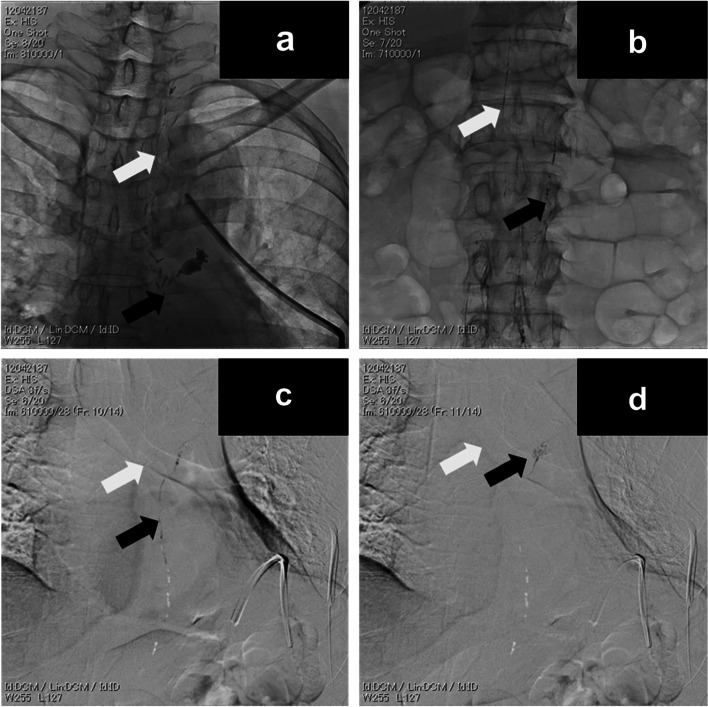
Fluoroscopic X-ray image during lymphangiography. **a** Two thoracic ducts (black arrow, white arrow) were observed. No connections were observed. **b** Chyle vessels (black arrow, white arrow) were present in the abdominal cavity. No cisterna chyli was observed. **c** Thoracic duct (black arrow). Left main bronchus (white arrow). **d** Leakage site from the thoracic duct running through the left thoracic cavity (black arrow). Left main bronchus (white arrow)

**Fig. 2:**
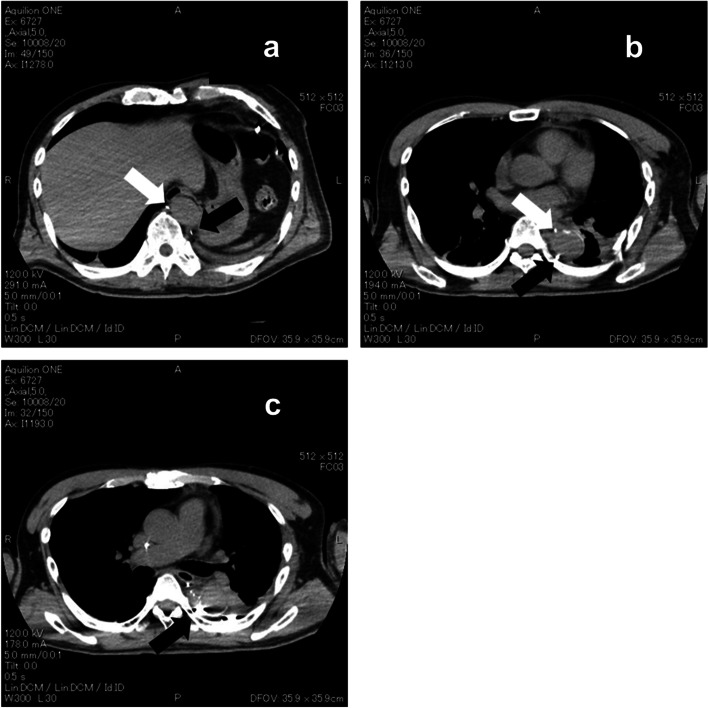
Chest computed tomography during lymphangiography. **a**, **b** Two thoracic ducts were observed (white arrow, black arrow). No connections were observed. **c** Leakage site from the thoracic duct running through the left thoracic cavity (black arrow)

**Fig. 3:**
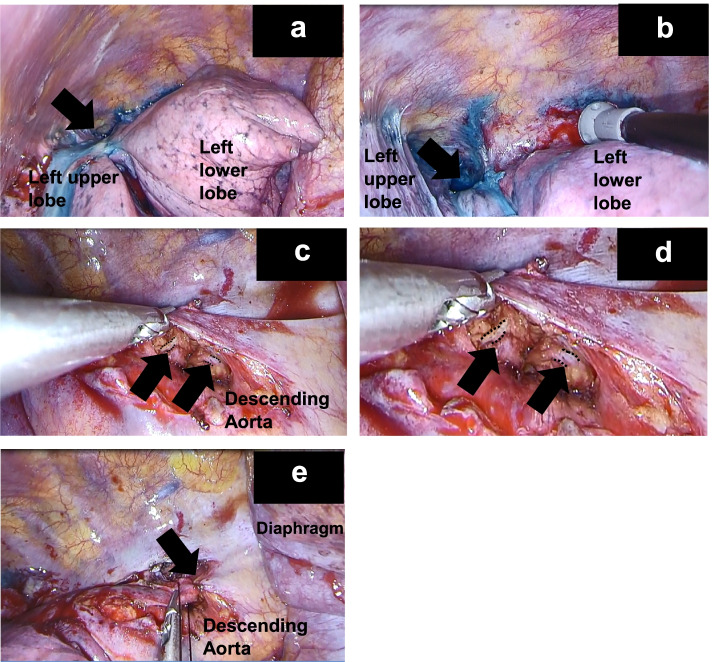
Intraoperative findings during video-assisted thoracic surgery. In all images, the cephalic direction is to the left and the caudal direction to the right. **a**, **b** Discharge of patent blue V was observed (black arrow). Lung adhesions were observed in the surrounding areas. **c**, **d** The thoracic duct running dorsally to the aorta was stained blue (black arrow, black dotted line). **d** is an enlarged version of **c**. **e** The thoracic duct was ligated to the surrounding connective tissue (black arrow)

A 69-year-old man underwent aortic replacement surgery using a prosthetic graft for an aortic aneurysm of the distal arch. On postoperative day 3, after he started oral intake, the pleural effusion from the thoracic drain became chylous, and he was diagnosed with chylothorax. The patient was treated with fasting and TPN, but the chylous pleural effusion continued at 500–1000 ml daily. Lymphangiography was performed on postoperative day 7 to identify the leakage site and TD course. The right inguinal lymph node was confirmed using ultrasound, punctured with a 23-G catheter needle (Terumo, Tokyo, Japan), and injected with iomeprol (Iomeron, Eisai, Tokyo, Japan). After confirming fluoroscopic contrast of the intra-abdominal chyle vessels, Lipiodol (Guerbet Japan, Tokyo, Japan) was injected into the lymph nodes. The fluoroscopic X-ray image showed two thoracic ducts without any connections (Fig. 1a). No cisterna chyli was observed in the abdominal cavity (Fig. 1b). The leakage site was observed in the thoracic duct running through the left thoracic cavity (Fig. 1c, d). A subsequent chest computed tomography scan showed similar findings (Fig. 2a–c). On postoperative day 8, octreotide acetate (Sun Pharma Japan Ltd., Tokyo, Japan) was administered. However, the chylous pleural effusion did not decrease. On postoperative day 21, we scheduled the patient for TDL. We used patent blue V (Sigma-Aldrich Japan GK, Tokyo, Japan) to identify the leakage site and TD course. In Japan, this is an off-label use; therefore, we received approval from the Ethics Committee of Fujita Health University Hospital (approval number: 09–8). After the induction of general anesthesia, 2 ml of 10% patent blue V mixed with 2 ml of iohexol (Omnipaque; GE Healthcare Japan, Tokyo, Japan) were injected into the inguinal lymph node at less than 0.8 ml/min under ultrasound guidance. Video-assisted thoracoscopic surgery was performed after confirming that the intra-abdominal chyle vessels were contrasted using fluoroscopic X-ray imaging. Thoracoscopy revealed patent blue V leakage in the left thoracic cavity; however, lung adhesions were observed in the surrounding area (Fig. 3a, b). While it was necessary to separate the adhesions to identify the leakage site, we decided against it to avoid lung injury. We identified a blue-colored TD in the dorsal aortic connective tissue (Fig. 3c, d), which were clipped using a DS clip (B. Braun Aesculap Japan Co., Ltd., Tokyo, Japan) (Fig. 3e). The operation time was approximately 120 min, and we could visualize the TD until the end of the operation. After the operation, blue pigmentation was observed on the patient's entire body and persisted until day 2. Oral intake was started on day 4, and the chylous pleural effusion did not increase. The thoracic drain was removed on day 6. Chylothorax did not recur and the patient was discharged from the hospital 14 days after the second surgery.

## Discussion and conclusions

Conservative treatment, such as a low-fat medium-chain triglyceride diet, fasting, and TPN, are treatment options for chylothorax [1–3]. Takuwa et al. reported that 23 out of 37 patients with chylothorax after lung cancer surgery were successfully treated with a low-fat diet [1]. However, the remaining 14 patients required pleurodesis or surgical intervention. In the present case, the patient was not successfully treated with fasting and TPN and ultimately underwent surgery.

Pleurodesis using multiple chemicals or autologous blood is one of the treatments for chylothorax [1–3]. Pleurodesis can control especially low levels of pleural effusion and was considered in our hospital because the pleural effusion was 500–1000 ml daily after fasting and TPN. However, pyothorax is a potential complication of pleurodesis, and since the patient had undergone aortic replacement with a prosthetic graft, pyothorax may have resulted in potentially fatal graft infection. In addition, adhesions in the thoracic cavity would have made subsequent TDL challenging. Therefore, pleurodesis was not performed.

Lymphatic embolization is another treatment option [3]. It requires cannulation of the catheter from the cisterna chyli, and advancing it to reach the leakage site. The patient had no cisterna chyli, precluding catheter insertion, and ruling out lymphatic embolization.

For the reasons above, we performed TDL. Administering milk or olives stains the TD white and allows for identification of its course and the leakage site [3, 4]. We chose patent blue V, because we believed that a blue stain would be more visible. As a result, a blue-stained TD on the dorsal side of the aorta appeared although we could not identify the leakage site because of lung adhesions. If milk or olives cannot be used due to allergies, this method might be a viable alternative.

Barthelmes et al. reported that the side effects of patent blue V when identifying sentinel lymph nodes in breast cancer were allergic reactions (0.85%), skin tattooing (0.012%), and a bluish hue persisting for a few hours (0.037%) [7]. In our case, blue pigmentation of the entire body persisted for 2 days after the second operation. However, we used 10% patent blue V compared to only 2.5% in Barthelmes et al.’s report. This significantly higher concentration may have caused the prolonged pigmentation.

Recently, ICG injection into lymph nodes or lymphatic vessels to identify the leakage site of chylous pleural effusion and the TD course has been reported [5, 6]. However, this method requires dedicated fluorescence imaging equipment, while one advantage of patent blue V is not requiring specific equipment.

We report a case of successful identification of the TD course using patent blue V. Our approach may be useful during TDL for chylothorax after thoracic surgery.

## Data Availability

All data generated or analyzed during this study are included in this published article.

## Abbreviations

TPN: Total parenteral nutrition
TDL: Thoracic duct ligation
TD: Thoracic duct
ICG: Indocyanine green
